# Hardy’s inequalities for the twisted convolution with Laguerre functions

**DOI:** 10.1186/s13660-017-1498-5

**Published:** 2017-09-15

**Authors:** Jinsen Xiao, Jianxun He

**Affiliations:** 1 School of Sciences, Guangdong University of Petrochemical Technology, Maoming, 525000 P.R. China; 2 School of Mathematics and Information Sciences, Guangzhou University, Guangzhou, 510006 P.R. China

**Keywords:** 43A85, 44A12, 52A38, Hardy’s inequality, special Hermite expansions, Laguerre function, Heisenberg group, Fourier transform

## Abstract

In this article, two types of Hardy’s inequalities for the twisted convolution with Laguerre functions are studied. The proofs are mainly based on an estimate for the Heisenberg left-invariant vectors of the special Hermite functions deduced by the Heisenberg group approach.

## Introduction

The classical Hardy’s inequalities on $\mathbb{C}$ state that if $f(z)=\sum_{k=0}^{\infty }a_{k}z^{k}$ belongs to the ordinary Hardy space $H^{p}(\mathbb{C})$, $0< p\leq 1$, then one has the following results for the coefficients:

$$ \vert a_{k} \vert \leq ck^{1/p-1}\Vert f \Vert _{H^{p}} $$

and

$$\begin{aligned} \sum_{k=0}^{\infty }(k+1)^{p-2}\vert a_{k} \vert ^{p}\leq c_{p}\Vert f \Vert _{H^{p}}^{p}, \end{aligned}$$

where $c_{p}$ depends only on *p* (see Theorems 6.2 and 6.4 in [1]).

The proofs of these inequalities are based on the atomic characterization of Hardy spaces. Using this idea, similar inequalities have been established for the discrete and continuous Fourier transforms [2–4], for Hermite expansions [5–9], for Laguerre expansions [6, 7, 10], and for special Hermite expansions [9, 11, 12]. More works related to this topic can be found in [13–16]. Now in this paper we continue to study Hardy’s inequalities for the twisted convolution with Laguerre functions defined by

$$ \varphi_{k}(z)=L_{k}^{n-1} \biggl(\frac{1}{2} \vert z \vert ^{2} \biggr)e^{-\frac{1}{4}\vert z \vert ^{2}}. $$

In [9], Radha and Thangavelu gave the following theorem, but the proof is left to the interested reader.

### Theorem 1.1

*Suppose* $f\in H_{a}^{p}(\mathbb{C}^{n})$ *with* $0< p\leq 1$. *Then there exists a constant* *c* *such that*

$$ \sum_{k=0}^{\infty } \Vert f\times \varphi_{k} \Vert _{2}^{p}(2k+n)^{- \sigma } \leq c\Vert f \Vert _{H_{a}^{p}(\mathbb{C}^{n})}^{p}, $$

*where* $\sigma = (\frac{n+1}{2} )(2-p)$.

Here $H_{a}^{p}(\mathbb{C}^{n})$ is the atomic Hardy space defined in terms of an atomic decomposition. When $p=1$, this inequality has been established by Thangavelu [12]. The key tool they provided to prove this result was the estimate for the ordinary differential of Laguerre functions

$$\begin{aligned} \sup_{z} \bigl\vert \partial_{z}^{\alpha } \varphi_{k}(z) \bigr\vert \leq ck ^{n+\vert \alpha \vert -1}. \end{aligned}$$

In this article we shall use an estimate related to the Heisenberg left-invariant vectors of the special Hermite functions, together with Taylor formula of Heisenberg group, to gain a new proof of this theorem. Moreover, we build another type of Hardy’s inequality.

### Theorem 1.2

*Suppose* $f\in H_{a}^{p}(\mathbb{C}^{n})$ *with* $0< p\leq 1$. *Then there exists a constant* *c* *such that*

$$ \Vert f\times \varphi_{k} \Vert _{2}\leq c(2k+n)^{\tau }\Vert f \Vert _{H_{a} ^{p}(\mathbb{C}^{n})}, $$

*where* $\tau ={ (n(\frac{2}{p}-1)-1 )/2}$.

Comparing Theorems 1.1 and 1.2 with the classical Hardy’s inequalities, we find that the varieties of indexes for $2k+n$ and $k+1$ have the same similarity. We shall show this fact in the last section.

The outline of this paper is as follows. In Section 2 we briefly summarize the harmonic analysis on the Heisenberg group and the atomic theory needed in the sequel. Section 3 is devoted to the proof of our main result. In order to do this, some lemmas are stated in this section. We will adopt the convention that *c* denotes constants which may be different from one statement to another.

## Preliminaries

The $(2n+1)$-dimensional Heisenberg group $\mathbb{H}^{n}$ is a Lie group structure on $\mathbb{C}^{n}\times \mathbb{R}$ with the multiplication law

$$\begin{aligned} (z,t) \bigl(z',t'\bigr)=\biggl(z+z',t+t'+ \frac{1}{2}\operatorname{Im}z\bar{z'}\biggr), \end{aligned}$$

where $z\bar{z'}=\sum_{j=1}^{n}z_{j}\bar{z'}_{j}$. The Lie algebra $\mathcal{G}$ of $\mathbb{H}^{n}$, which admits a stratification by $\mathcal{G}=V_{1}\oplus V_{2}$, is generated by the left-invariant vector fields

$$ Z_{j}=\frac{\partial }{\partial z_{j}}+\frac{i}{4} \bar{z}_{j} \frac{ \partial }{\partial t},\qquad \bar{Z}_{j}=\frac{\partial }{\partial \bar{z} _{j}}-\frac{i}{4} z_{j}\frac{\partial }{\partial t}, \quad 1\leq j\leq n, $$

and $T=\frac{\partial }{\partial t}$. The horizontal layer is just the first layer $V_{1}$ generated by $Z_{j}, \bar{Z}_{j}, 1\leq j\leq n$.

Now let $\sigma =(\sigma_{1},\ldots ,\sigma_{2n},\sigma_{2n+1})=(1, \ldots ,1,2)$, $I=(i_{1},\ldots ,i_{k})$ with $i_{1},\ldots ,i_{k} \in \{1,2,\ldots ,2n+1\}$, and use the notation $\sigma (I)= \sigma_{i_{1}}+\cdots +\sigma_{i_{k}} $ to denote the homogeneous length of *I*. We also write $X_{j}=Z_{j}$, $X_{n+j}=\bar{Z}_{j}, 1\leq j \leq n$, $X_{2n+1}=T$ and use the notation $X_{I}=X_{i_{1}}\cdots X _{i_{k}}$ to denote the left-invariant differential operator.

A function *P* on $\mathbb{H}^{n}$ is called a polynomial if $P\circ \exp $ is a polynomial on $\mathcal{G}$. Every polynomial on $\mathbb{H}^{n}$ can be written uniquely as a finite sum

$$ P=\sum_{J}a_{J}\eta^{J},\quad a_{J}\in \mathbb{C}, \eta^{J}=\eta_{1}^{j _{1}} \cdots \eta_{2n+1}^{j_{2n+1}}, $$

where $\eta_{j}=\zeta_{j}\circ \log $, $\zeta_{1},\ldots ,\zeta_{2n+1}$ is the basis for $\mathcal{G}^{*}$ dual to the basis $X_{1},\ldots ,X _{2n+1}$ for $\mathcal{G}$. The monomial $\eta^{J}$ is homogeneous of degree $d(J)=\sum_{i=1}^{2n}j_{i}+2j_{2n+1}$ and the homogeneous degree of *P* is given by $d(P)=\max \{d(J):a_{J}\neq 0\}$. For $s\in \mathbb{N}=\{0,1,2,\ldots \}$, we denote by $\mathscr{P}_{s}$ the space of polynomials whose homogeneous degree ≤*s*. Let $f\in C^{s+1}( \mathbb{H}^{n})$, the left Taylor polynomial of *f* at $(z,t)\in \mathbb{H}^{n}$ of homogeneous degree *s* is the unique $P_{s}(f,(z,t)) \in \mathscr{P}_{s}$ such that $X_{I}P_{s}(f,(z,t))(0)=X_{I}f(z,t)$ for $\sigma (I)\leq s$. Let $(z',t'), (z,0)\in \mathbb{H}^{n}$ and suppose that $f\in C^{k+1} (k\in \mathbb{N})$, then by Bonfiglioli [17] one has the following horizontal Taylor formula with integral remainder for the Heisenberg group:

$$\begin{aligned} f \bigl(\bigl(z',t'\bigr) (z,0) \bigr)&=f \bigl(z',t'\bigr)+\sum_{l=1}^{k} \mathop{\sum_{I=(i_{1},\ldots ,i_{l})}}_{i_{1},\ldots ,i_{l}\leq 2n}\frac{X _{I}f(z',t')}{l!}\xi_{i_{1}}\cdots \xi_{i_{l}} \\ &\quad {}+\mathop{\sum_{I=(i_{1},\ldots ,i_{k+1})}}_{i_{1},\ldots ,i_{k+1}\leq 2n} \xi_{i_{1}}\cdots \xi_{i_{k+1}} \int_{0}^{1}(X_{I}f) \biggl( \bigl(z',t'\bigr) \cdot \exp \biggl(\sum _{j\leq 2n}s\xi_{j}X_{j} \biggr) \biggr) \frac{(1-s)^{k}}{k!}\,ds, \end{aligned}$$

where the notation $\log (z,0)=\sum_{j\leq 2n}\xi_{j}X_{j}$.

Now we pick up the orthonormal basis in $L^{2}(\mathbb{R}^{n})$ to be the Hermite functions given by

$$ \Phi_{\alpha }(\xi )=\bigl(2^{\vert \alpha \vert }\alpha !\sqrt{\pi } \bigr)^{-1/2}e^{-\vert \xi \vert ^{2}/2}\prod_{j=1}^{n}H_{\alpha_{j}}( \xi_{j}), $$

where $H_{k}(s)=(-1)^{k} e^{s^{2}} (\frac{d}{ds} )^{k}(e^{-s ^{2}})$ is the Hermite polynomial. For $\lambda \in \mathbb{R}\backslash \{0\}$, the Schrödinger representation $\Pi_{\lambda }$ of $\mathbb{H}^{n}$ acts on $L^{2}(\mathbb{R}^{n})$ by

$$\begin{aligned} \Pi_{\lambda }(z,t)\varphi (\xi )=e^{i\lambda t} e^{i\lambda (x\cdot \xi +\frac{1}{2}x\cdot y)}\varphi ( \xi +y), \end{aligned}$$

where $z=x+iy$. Moreover, $\Pi_{\lambda }(z,t)$ is a strongly continuous unitary representation satisfying

$$ \Pi_{\lambda }(z,t)\Pi_{\lambda }\bigl(z',t' \bigr)=\Pi_{\lambda } \bigl((z,t) \bigl(z',t'\bigr) \bigr), $$

and $\Pi_{\lambda }(z,t)\varphi $ converges to *φ* in $L^{2}(\mathbb{R}^{n})$ as $(z,t)\rightarrow 0$ in $\mathbb{H}^{n}$. If we set $\Phi_{\alpha , \beta }(z,t)= (\Pi_{1}(z,t)\Phi_{\alpha }, \Phi_{\beta } )$ and $\Pi (z)=\Pi_{1}(z,0)$, then $\Phi_{\alpha , \beta }(z)=\Phi_{\alpha , \beta }(z,0)= (\Pi (z) \Phi_{\alpha },\Phi_{\beta } )$ is called the special Hermite function and $\{\Phi_{\alpha , \beta }(z)\}$ forms an orthonormal basis for $L^{2}(\mathbb{C}^{n})$. Note that the operator Π is an irreducible projective representation of $\mathbb{C}^{n}$ into $L^{2}(\mathbb{R}^{n})$ such that

$$ \Pi (z+w)=\Pi (z)\Pi (w)e^{-\frac{i}{2}\operatorname{Im}z\bar{w}}, $$

and the integral

$$ \int_{\mathbb{C}^{n}}f(z)\Pi (z)\,dz $$

is nothing but the Weyl transform of *f*. Now suppose $f\in L^{2}( \mathbb{C}^{n})$, one has the expansion

$$ f(z)=\sum_{\alpha \in \mathbb{N}^{n}}\sum_{\beta \in \mathbb{N}^{n}} (f,\Phi_{\alpha , \beta } )\Phi_{\alpha , \beta }(z), $$

from which, together with the relation of the special Hermite function and the Laguerre function

$$ \sum_{\vert \alpha \vert =k}\Phi_{\alpha ,\alpha }(z)=(2\pi )^{-\frac{n}{2}} \varphi_{k}(z) $$

and the twisted convolution of functions *f* and *g*

$$ f\times g(z)= \int_{\mathbb{C}^{n}}f(z-w)g(w)e^{\frac{i}{2}\operatorname{Im}(z \bar{w})}\,dw, $$

one can rewrite the special Hermite expansion as

$$ f(z)=(2\pi )^{-n}\sum_{k=0}^{\infty } f \times \varphi_{k}(z). $$

For various results related to these expansions, readers can refer to [18, 19].

Now we are going to introduce the atomic Hardy spaces $H_{a}^{p}( \mathbb{C}^{n})$. For $0< p\leq 1\leq q\leq \infty , p\neq q$, $s\in \mathbb{N}$ and $s\geq [2n(1/p-1)]$, a function *a* is called the $(p,q,s)$-atom with the center $z_{0}$ if it satisfies (i) $\operatorname{supp}(a)\subset B_{r}(z_{0})$, (ii) $\Vert a \Vert _{q}\leq \vert B_{r}(z_{0}) \vert ^{1/q-1/p}$, and (iii) the cancelation conditions $\int_{\mathbb{C}^{n}}a(z)P(z-z_{0})e^{\frac{i}{2} \operatorname{Im}z\bar{z}_{0}}\,dz=0$ for any polynomial *P* whose degree ≤*s*.

For $0< p\leq 1$, a tempered distribution *f* is said to be an element of the atomic Hardy space $H_{a}^{p}(\mathbb{C}^{n})$ if it can be characterized by the decomposition

$$ f(z)=\sum_{j=1}^{\infty }\lambda_{j}a_{j}(z), $$

where $a_{j}$’s are $(p,q,s)$-atoms and $\sum_{j=1}^{\infty }\vert \lambda_{j} \vert ^{p}<\infty $. Moreover, the space $H_{a}^{p}(\mathbb{C} ^{n})$ can be made into a metric space by means of the quasi-norm defined by

$$ \Vert f \Vert _{H_{a}^{p}}^{p}=\inf \Biggl\{ \sum_{j=1}^{\infty }\vert \lambda_{j} \vert ^{p}: f=\sum_{j=1}^{\infty } \lambda_{j}a_{j}\Biggr\} . $$

Note that the cancelation condition of $(p,q,s)$-atom is defined in consideration of the Weyl transform, and thus the atomic Hardy space $H_{a}^{p}(\mathbb{C}^{n})$ defined above is different from the ordinary Hardy space $H^{p}(\mathbb{C}^{n})$ for $0< p<1$ (see [9]).

## Proofs of the main results

We are now in a position to give the proofs of the main results. First we state some crucial lemmas.

### Lemma 3.1

*For any* $(z,t)\in \mathbb{H}^{n}$ *and* $\alpha \in \mathbb{N}^{n}$, *we have*

$$\begin{aligned} \sum_{\beta \in \mathbb{N}^{n}} \bigl\vert X_{I} \Phi_{\alpha ,\beta }(z,t) \bigr\vert ^{2}\leq c_{I} \bigl(2 \vert \alpha \vert +n \bigr)^{\sigma (I)}. \end{aligned}$$

### Proof

Expanding $\Pi_{1}(z,t)\Phi_{\alpha }(\xi )$ in terms of $\Phi_{ \beta }$ by

$$ \Pi_{1}(z,t)\Phi_{\alpha }(\xi )=\sum _{\beta } \bigl(\Pi_{1}(z,t) \Phi_{\alpha }, \Phi_{\beta } \bigr)\Phi_{\beta }(\xi ), $$

we get

$$\begin{aligned} \Phi_{\alpha ,\alpha } \bigl((-z,-t) (z,t) \bigr) =& \bigl(\Pi_{1}(z,t) \Phi_{\alpha }, \Pi_{1}(z,t)\Phi_{\alpha } \bigr) \\ =&\sum_{\beta \in \mathbb{N}^{n}} \bigl(\Pi_{1}(z,t) \Phi_{\alpha }, \Phi_{\beta } \bigr) \bigl(\Phi_{\beta }, \Pi_{1}(z,t)\Phi_{\alpha } \bigr) \\ =&\sum_{\beta \in \mathbb{N}^{n}} \bigl\vert \Phi_{\alpha ,\beta }(z,t) \bigr\vert ^{2}, \end{aligned}$$

from which together with the facts that $\Phi_{\alpha ,\alpha }((-z,-t)(z,t))= \Phi_{\alpha ,\alpha }(0,0)=1$ and for $1\leq j\leq n$,

$$\begin{aligned}& Z_{j}\Phi_{\alpha ,\beta }(z,t) = i (2\alpha_{j}+2 )^{1/2} \Phi_{\alpha +e_{j},\beta }(z,t), \\& \bar{Z}_{j}\Phi_{\alpha ,\beta }(z,t) = i (2\alpha_{j} )^{1/2} \Phi_{\alpha -e_{j},\beta }(z,t), \end{aligned}$$

where $\{e_{1},\ldots ,e_{n}\}$ is the standard basis of $\mathbb{R} ^{n}$, we then deduce this lemma. □

### Lemma 3.2

*Let* $P_{k} {(\Phi_{\alpha ,\gamma },0)}$ *be the horizontal Taylor polynomial of* $\Phi_{\alpha ,\gamma }$ *at the origin of homogeneous degree k*. *Then*, *for* $(z,0),(z_{0},0)\in \mathbb{H}^{n}$, *we have*

$$\begin{aligned} \sum_{\beta \in \mathbb{N}^{n}} \biggl\vert \sum_{\gamma }\Phi_{\gamma ,\beta }(z_{0},0) \bigl(\Phi_{\alpha ,\gamma }(z,0)-P_{k} {(\Phi_{\alpha ,\gamma },0)}(z,0) \bigr)\biggr\vert ^{2} \leq c_{k} \vert z \vert ^{2k+2} \bigl(2\vert \alpha \vert +n \bigr)^{k+1}. \end{aligned}$$

### Proof

For the special Hermite functions, we find that

$$\begin{aligned} \Phi_{\alpha ,\beta }(z_{0}+z)=\sum_{\gamma } \Phi_{\gamma ,\beta }(z _{0})\Phi_{\alpha ,\gamma }(z)e^{\frac{i}{2}\operatorname{Im}z\bar{z}_{0}}. \end{aligned}$$

Hence

$$\begin{aligned} \biggl\vert \sum_{\gamma }\Phi_{\gamma ,\beta }(z_{0},0)X_{I} \Phi_{\alpha ,\gamma }(z,0) \biggr\vert = \bigl\vert X_{I} \Phi_{\alpha ,\beta }\bigl((z_{0},0) (z,0)\bigr) \bigr\vert . \end{aligned}$$

Then, for $(z,0)=\exp (\sum_{j\leq 2n}\xi_{j}X_{j})$, by the horizontal Taylor formula and Lemma 3.1, we have

$$\begin{aligned}& \sum_{\beta \in \mathbb{N}^{n}} \biggl\vert \sum_{\gamma }\Phi_{\gamma ,\beta }(z_{0}) \bigl( \Phi_{\alpha ,\gamma }(z,0)-P_{k} {(\Phi_{\alpha ,\gamma },0)}(z,0) \bigr) \biggr\vert ^{2} \\& \quad = \sum_{\beta \in \mathbb{N}^{n}} \biggl\vert \sum_{\gamma }\Phi_{\gamma ,\beta }(z_{0}) \biggl( \mathop{\sum_{I=(i_{1},\ldots ,i_{k+1})}}_{i_{1},\ldots ,i_{k+1}\leq 2n}\xi_{i_{1}}\cdots \xi_{i_{k+1}} \\& \quad \quad {}\times\int_{0}^{1}X_{I}\Phi_{\alpha ,\gamma } \biggl(\exp \biggl(\sum_{j\leq 2n}s\xi_{j}X_{j} \biggr) \biggr)\frac{(1-s)^{k}}{k!}\,ds \biggr) \biggr\vert ^{2} \\& \quad \leq \sum_{\beta \in \mathbb{N}^{n}}\mathop{\sum _{I=(i_{1},\ldots ,i_{k+1})}}_{i_{1},\ldots ,i_{k+1}\leq 2n} \vert \xi_{i_{1}}\cdots \xi_{i_{k+1}} \vert ^{2} \\& \quad \quad {}\times \mathop{\sum_{I=(i_{1},\ldots ,i_{k+1})}}_{i_{1},\ldots ,i_{k+1}\leq 2n} \biggl\vert \int_{0}^{1}\sum_{\gamma } \Phi_{\gamma ,\beta }(z_{0},0)X_{I}\Phi_{\alpha ,\gamma } \biggl(\exp \biggl(\sum_{j\leq 2n}s\xi_{j}X_{j} \biggr) \biggr)\frac{(1-s)^{k}}{k!}\,ds \biggr\vert ^{2} \\& \quad \leq c'_{k}\vert z \vert ^{2k+2} \mathop{\sum_{I=(i_{1},\ldots ,i_{k+1})}}_{i_{1},\ldots ,i_{k+1}\leq 2n} \int_{0}^{1}\sum_{\beta \in \mathbb{N}^{n}} \bigl\vert X_{I}\Phi_{\alpha ,\beta }\bigl((z_{0},0) (z_{s},0)\bigr) \bigr\vert ^{2}\,ds \int_{0}^{1}\frac{(1-s)^{2k}}{(k!)^{2}}\,ds \\& \quad \leq c_{k}\vert z \vert ^{2k+2} \bigl(2\vert \alpha \vert +n \bigr)^{k+1}, \end{aligned}$$

where we have used the notation $(z_{s},0)=\exp (\sum_{j\leq 2n}s\xi _{j}X_{j})$ and the fact $\vert \xi_{i_{l}} \vert \leq c\vert (z,0) \vert =c\vert z \vert $ (see Lemma 3 of [17]) in the second inequality. □

### Lemma 3.3

*Suppose that* *f* *is a* $(p,2,s)$-*atom supported in a ball* $B_{r}(z_{0})$. *Then we have*

$$ \Vert f\times \varphi_{k} \Vert _{2}^{2}\leq c(2k+n)^{s+n}\Vert f \Vert _{2} ^{2-\frac{s+1+n}{n(1/p-1/2)}}. $$

### Proof

From p.58 of [19] we see that

$$\begin{aligned} f\times \varphi_{k}(z)=(2\pi )^{n}\sum _{\vert \alpha \vert =k} \sum_{\beta \in \mathbb{N}^{n}} (f, \Phi_{\alpha , \beta } ) \Phi_{\alpha , \beta }(z). \end{aligned}$$

It follows that

$$ \Vert f\times \varphi_{k} \Vert _{2}^{2}=(2\pi )^{2n}\sum_{\vert \alpha \vert =k}\sum _{\beta \in \mathbb{N}^{n}} \bigl\vert (f,\Phi_{\alpha , \beta } ) \bigr\vert ^{2}. $$

Now let $P_{s} {(\Phi_{\alpha ,\gamma },0)}(z,0)$ be the Taylor polynomial of $\Phi_{\alpha ,\gamma }(z,0)$ at the origin of homogeneous degree *s*. Then, by the cancelation condition of *p*-atom and Lemma 3.2, we have

$$\begin{aligned}& \sum_{\vert \alpha \vert =k}\sum_{\beta \in \mathbb{N}^{n}} \bigl\vert (f,\Phi_{\alpha , \beta } ) \bigr\vert ^{2} \\& \quad = \sum_{\vert \alpha \vert =k}\sum_{\beta } \biggl\vert \int_{B_{r}(0)} \bar{f}(z_{0}+z) \Phi_{\alpha ,\beta }(z_{0}+z) \,dz \biggr\vert ^{2} \\& \quad = \sum_{\vert \alpha \vert =k}\sum_{\beta } \biggl\vert \int_{B_{r}(0)}\bar{f}(z_{0}+z)\sum _{\gamma }\Phi_{\gamma ,\beta }(z_{0}) \Phi_{\alpha ,\gamma }(z)e^{\frac{i}{2}\operatorname{Im}z\bar{z}_{0}}\,dz \biggr\vert ^{2} \\& \quad = \sum_{\vert \alpha \vert =k}\sum_{\beta } \biggl\vert \sum_{\gamma }\Phi_{\gamma ,\beta }(z_{0}) \int_{B_{r}(0)}\bar{f}(z_{0}+z) \bigl( \Phi_{\alpha ,\gamma }(z,0)-P_{s} {(\Phi_{\alpha ,\gamma },0)}(z,0) \bigr)e^{\frac{i}{2}\operatorname{Im}z\bar{z}_{0}}\,dz \biggr\vert ^{2} \\& \quad \leq \sum_{\vert \alpha \vert =k} \int_{B_{r}(0)} \bigl\vert \bar{f}(z_{0}+z) \bigr\vert ^{2}\,dz \sum_{\beta } \int_{B_{r}(0)} \biggl\vert \sum_{\gamma } \Phi_{\gamma ,\beta }(z_{0}) \bigl(\Phi_{\alpha ,\gamma }(z,0)-P_{s}{( \Phi_{\alpha ,\gamma },0)}(z,0) \bigr) \biggr\vert ^{2}\,dz \\& \quad \leq c\sum_{\vert \alpha \vert =k}\Vert f \Vert _{2}^{2} \bigl(2\vert \alpha \vert +n \bigr)^{s+1} \int_{B_{r}(0)}\vert z \vert ^{2s+2}\,dz \\& \quad \leq c(2k+n)^{s+n}\Vert f \Vert _{2}^{2-\frac{2s+2+2n}{2n(1/p-1/2)}}, \end{aligned}$$

where in the last inequality we have used (ii) of *p*-atom. □

### Proof of Theorem 1.1

For $f\in H_{a}^{p}(\mathbb{C} ^{n})$, it follows that $f=\sum_{j=1}^{\infty }\lambda_{j}a_{j}$, where $a_{j}$’s are $(p,2,s)$-atoms supported in $B_{r}(z_{0})$. Then we get

$$\begin{aligned} \sum_{k=0}^{\infty } \Vert f\times \varphi_{k} \Vert _{2}^{p}(2k+n)^{- \sigma } \leq &\sum_{k=0}^{\infty }\sum _{j=1}^{\infty }\vert \lambda_{j} \vert ^{p} \Vert a_{j}\times \varphi_{k} \Vert _{2}^{p}(2k+n)^{-\sigma } \\ \leq & \sum_{j=1}^{\infty }\vert \lambda_{j} \vert ^{p} \biggl(\sum _{2k+n>r^{-2}}+ \sum_{2k+n\leq r^{-2}} \biggr)\Vert a_{j}\times \varphi_{k} \Vert _{2}^{p}(2k+n)^{- \sigma } \\ =&\sum_{j=1}^{\infty }\vert \lambda_{j} \vert ^{p}(S_{1}+S_{2}), \end{aligned}$$

from which we see that, to get this theorem, it suffices to show that $S_{1}$ and $S_{2}$ are bounded.

For the term $S_{1}$, Hölder’s inequality gives

$$\begin{aligned} S_{1} =& \sum_{2k+n>r^{-2}}\Vert a_{j}\times \varphi_{k} \Vert _{2}^{p}(2k+n)^{- \sigma } \\ \leq & \biggl(\sum_{2k+n>r^{-2}}\Vert a_{j} \times \varphi_{k} \Vert _{2}^{2} \biggr)^{\frac{p}{2}} \biggl(\sum_{2k+n>r^{-2}}(2k+n)^{- \frac{2\sigma }{2-p}} \biggr)^{\frac{2-p}{2}} \\ \leq & c \Vert a_{j} \Vert _{2}^{p}r^{2\sigma +p-2} \\ \leq & c r^{np-2n} r^{2\sigma +p-2} \\ =& c, \end{aligned}$$

where the last inequality follows from the fact $\Vert a_{j} \Vert _{2}\leq cr ^{2n(\frac{1}{2}-\frac{1}{p})}$.

Now, for the term $S_{2}$, by Lemma 3.3, we have

$$\begin{aligned} S_{2} =& \sum_{2k+n\leq r^{-2}}\Vert a_{j}\times \varphi_{k} \Vert _{2}^{p}(2k+n)^{- \sigma } \\ \leq & \Vert a_{j} \Vert _{2}^{p(1-\frac{s+1+n}{2n(1/p-1/2)})} \sum _{2k+n\leq r^{-2}}(2k+n)^{-\sigma +(s+n)p/2} \\ \leq & c r^{(s+1)p-2n+2np} r^{2\sigma -(s+n)p-2} \\ =& c. \end{aligned}$$

The proof of this theorem is completed. □

### Proof of Theorem 1.2

Again assume $f=\sum_{j=1}^{ \infty }\lambda_{j}a_{j}$, where $a_{j}$’s are $(p,2,s)$-atoms supported in $B_{r}(z_{0})$, then

$$\begin{aligned} \Vert f\times \varphi_{k} \Vert _{2} \leq &\sum _{j=1}^{\infty }\vert \lambda_{j} \vert \Vert a_{j}\times \varphi_{k} \Vert _{2}. \end{aligned}$$

In view of this, it is enough to show that

$$ \Vert a_{j}\times \varphi_{k} \Vert _{2}\leq c (2k+n)^{ (n(\frac{2}{p}-1)-1 )/2}. $$

Firstly, if $r^{-2}\leq 2k+n$, by the property (ii) of *p*-atom, we have

$$\begin{aligned} \Vert a_{j}\times \varphi_{k} \Vert _{2}^{2} =& c \sum_{\vert \alpha \vert =k} \sum _{\beta \in \mathbb{N}^{n}} \bigl\vert (a_{j}, \Phi_{\alpha , \beta } ) \bigr\vert ^{2} \\ \leq & c \sum_{\vert \alpha \vert =k} \sum _{\beta \in \mathbb{N}^{n}} \int_{B _{r}}\bigl\vert \Phi_{\alpha ,\beta }(z) \bigr\vert ^{2}\,dz \Vert a_{j} \Vert _{2}^{2} \\ \leq & cr^{2n} \Vert a_{j} \Vert _{2}^{2} \sum_{\vert \alpha \vert =k}1 \\ \leq & cr^{2n+4n(\frac{1}{2}-\frac{1}{p})} (2k+n)^{n-1} \\ \leq & c(2k+n)^{n(\frac{2}{p}-1)-1}. \end{aligned}$$

Now, if $r^{-2}>2k+n$, then by Lemma 3.3 we get

$$\begin{aligned} \Vert a_{j}\times \varphi_{k} \Vert _{2}^{2} \leq & c(2k+n)^{s+n}\Vert a_{j} \Vert _{2}^{2-\frac{s+1+n}{n(1/p-1/2)}} \\ \leq & c(2k+n)^{s+n} r^{-2(2n(\frac{1}{p}-1)-s-1)} \\ \leq & c(2k+n)^{n(\frac{2}{p}-1)-1}. \end{aligned}$$

This ends the proof of this theorem. □

## Conclusion and remark

In this paper we have used the Heisenberg method to prove two Hardy’s inequalities for the twisted convolution with Laguerre functions. We also note that the two classical Hardy’s inequalities can be written as

$$\begin{aligned} (k+1)^{p-1}\vert a_{k} \vert ^{p}\leq c\Vert f \Vert _{H^{p}}^{p} \end{aligned}$$

and

$$\begin{aligned} \sum_{k=0}^{\infty }(k+1)^{p-1-1}\vert a_{k} \vert ^{p}\leq c\Vert f \Vert _{H^{p}}^{p}. \end{aligned}$$

Obviously, the difference of the powers of $k+1$ in these two inequalities is 1. At present situation, our results show that

$$\begin{aligned} (2k+n)^{ (\frac{n+1}{2} )(2-p)-1}\Vert f \times \varphi_{k} \Vert _{2} ^{p}\leq C_{p}\Vert f \Vert ^{p}_{H_{a}^{p}({\mathbb{C}}^{n})} \end{aligned}$$

and

$$\begin{aligned} \sum_{k=0}^{\infty }(2k+n)^{ (\frac{n+1}{2} )(2-p)}\bigl\Vert f \times\varphi_{k}\bigl\Vert _{2}^{p}\leq C_{p} \bigr\Vert f \bigr\Vert ^{p}_{H_{a}^{p}({\mathbb{C}} ^{n})}, \end{aligned}$$

which indicate that the difference of the powers for $2k+n$ is also 1.
